# Phenotypic heterogeneity of capsule production across opportunistic pathogens

**DOI:** 10.1128/mbio.01807-25

**Published:** 2025-09-04

**Authors:** Amandine Nucci, Julie Le Bris, Sara Diaz-Diaz, Lilibeth Torres-Elizalde, Eduardo P. C. Rocha, Olaya Rendueles

**Affiliations:** 1 Institut Pasteur, CNRS UMR3525, Microbial Evolutionary Genomics, Université Paris Cité 555089, Paris, France; 2 Collège Doctoral, École Doctorale Complexité du Vivant, Sorbonne Université 27063, Paris, France; 3 Laboratoire de Microbiologie et Génétique Moléculaires (LMGM), CNRS UMR5100, Centre de Biologie Intégrative (CBI), CNRS, Université de Toulouse 27091, Toulouse, France; National University of Singapore, Singapore, Singapore

**Keywords:** phage resistance, virulence, commensalism, host-pathogen interactions, epigenetics

## Abstract

Phenotypic heterogeneity allows bacteria to adapt fast to changing environments. Extracellular capsules are well-known virulence factors, but also increase the cell adaptability and prevalence under hostile conditions. To limit their cost, some species regulate capsule production by genetic phase variation. Here, we demonstrated that phenotypic heterogeneity is a major mechanism controlling capsule production in *Klebsiella* and *Acinetobacter* species. We designed a method to agnostically measure heterogeneity and show that 71% of *Klebsiella pneumoniae* strains can be heterogeneous. This is mostly associated with *K. pneumoniae* strains that do not encode *rmp*, a genetic determinant of hypervirulence. Capsule serotype exchanges across several genetic backgrounds revealed that heterogeneity depends on specific genome-capsule locus interactions. Importantly, we showed that heterogeneity provides a fitness advantage especially in conditions where the capsule is costly, as estimated by comparing non-heterogeneous and heterogeneous strains during competition with their non-capsulated variants. Finally, heterogeneity impacts phage adsorption patterns, and could thus alter the rate of horizontal gene transfer events. This unsuspected heterogeneity may help understand the transition from commensalism to pathogenesis and can have important implications in virulence, environmental survival and evolution of some ESKAPE pathogens.

**IMPORTANCE:**

The polysaccharidic capsule is present in ~50% of species across the bacterial phylogeny, including all ESKAPE microorganisms, the six most significant multidrug-resistant (MDR) nosocomial pathogens. It is also an important virulence factor and a major target for both phage therapy and the development of vaccines. Here, we reveal that in two major genera of ESKAPE pathogens, *Klebsiella* spp. and *Acinetobacter* spp., capsule production within clonal populations is heterogeneous, leading to mixed populations of hyper-, hypo-, and intermediate-capsulated cells. Such heterogeneity responds to different environmental cues, including changes in nutrient availability and spatial structure. We show that this plasticity, known to enable faster, more efficient adaptation to environmental changes, limits capsule costs and could explain *Klebsiella* and *Acinetobacter* resilience. Finally, capsule heterogeneity can play a major role in bacterial evolution, as a driver of horizontal gene transfer, and in treatment failure. Thus, it should be taken into account in the design of prophylactic strategies and antimicrobial therapy.

## INTRODUCTION

Phenotypic heterogeneity is defined as the difference in a particular trait between genetically identical cells growing in a homogenous environment (1, 2). It can provide a significant advantage in fluctuating environments as it can increase the survival of cells. Both natural competence (3) and antibiotic persistence (4) are well-known traits subjected to phenotypic heterogeneity. Most often, heterogeneity results in two distinct subpopulations displaying or not displaying the specific trait (5). However, other traits, such as cell size, are also heterogeneous, but rather than discrete subpopulations, the observed heterogeneity follows a continuous distribution (6).

The bacterial extracellular capsule is present in ~50% of species across the bacterial phylogeny (7). All ESKAPE microorganisms, the six most significant multidrug-resistant (MDR) nosocomial pathogens, produce a capsule or capsule-like polysaccharides. It is considered a major virulence factor that limits the host’s immune response (8) but also a major defense against T6SS-mediated attacks (9). The capsule is also a key factor driving horizontal gene transfer, and thus, plays a central role in adaptation and genomic evolution across different species, including *Streptococcus pneumoniae* (10) and *Klebsiella pneumoniae* (11, 12). Capsule production is generally considered a costly trait, and different bacteria have evolved strategies to limit its cost. The sialic acid capsule of *Neisseria meningitidis* (13) is controlled by a phase variation mechanism, which generates population heterogeneity due to genetic changes. The insertion and deletion of cytosine residues in a stretch of seven cytosines located in the *siaD* gene (13) results in *ON/OFF* phase variation. Such bet-hedging genotypes are also easily evolvable in fluctuating environments, as shown in *Pseudomonas fluorescens* (14), whereby an epigenetic switch dependent on a *carB* mutation led to bistable ON/OFF colonies (15). Finally, in uropathogenic *Escherichia coli* encoding group 2 capsules, phenotypic heterogeneity has been documented when growing in urine (16).

*Klebsiella* and *Acinetobacter* species are environmental and clinically important bacteria. They comprise opportunistic pathogens that cause nosocomial and community-acquired infections, such as pneumonia and urinary tract infections (17–19). They possess a large group 1 capsule, which is also known to become hypermucoid (20, 21), and it is the main receptor for bacteriophages (22–24). In *K. pneumoniae*, capsule regulation is remarkably complex (21), with over one hundred genes involved (25, 26). Regulation is strain-dependent (25) and also relies on the resident mobile genetic elements, namely the hypervirulent plasmid encoding *rmp* locus (27–29) and *iroP* (30). Recently, mutations in the promoter of *rmp* have been shown to lead to a switch in capsulation (31, 32), similar to the phase variation described in other species. However, these only affect a reduced subset of strains (i.e., those carrying the hypervirulent plasmid). It is also known that in nutrient-rich environments, capsule production is costly, resulting in 20% of fitness reduction (33). This results in the fast emergence of non-capsulated variants, mostly due to accumulation of mutations in the first gene of the biosynthesis chain of the capsule (11). However, these mutations are not easily reversible. Despite the existence of mechanisms to adapt capsule expression to different environmental cues, it remained puzzling that such a versatile and ubiquitous bacterium colonizing different niches and with different eco-pathological lifestyles, i.e., from soil to commensalism to infection of diverse body sites, does not display a switching mechanism. We thus posit that *K. pneumoniae* capsules could be under a finer population heterogeneity.

Here, we designed agnostic methodologies to systematically analyze the distribution of capsule amount within genetically identical cells. We reveal the existence of a novel mechanism of phenotypic heterogeneity in the production of the bacterial capsule in different *Klebsiella* that does not depend on a mutational switch. We show that heterogeneity robustly responds to changes in the environment and is partly determined by the capsule locus sequence. The described heterogeneity is associated with strains lacking either the hypervirulence plasmid or the *rmp* locus, the so-called classical *K. pneumoniae*. Our results also show that heterogeneity limits the cost in environments in which the capsule is known to be a burden. Finally, this phenomenon is not constricted to the *Klebsiella* genus but is also present in *Acinetobacter* species and could be a widespread mechanism across ESKAPE pathogens. Our results may provide clues to the transition from commensalism to pathogenesis and may be relevant to consider when developing capsule-targeting vaccines.

## RESULTS

### Quantification of capsule phenotypic heterogeneity

**Fig 1:**
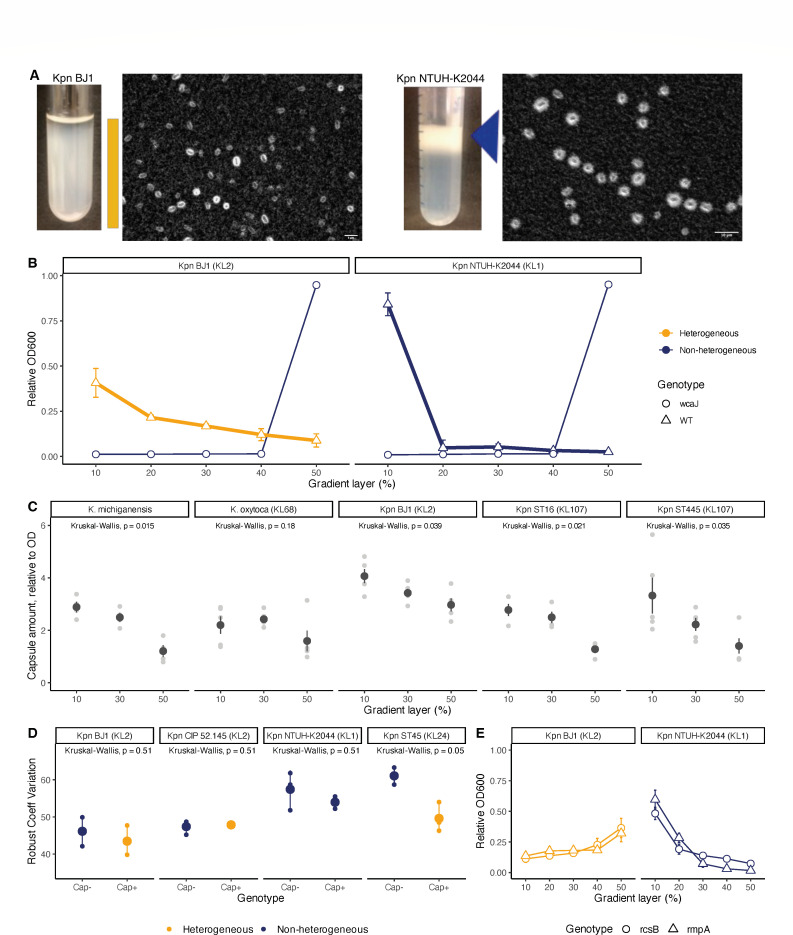
Capsule heterogeneity in *Klebsiella* spp. (A) Microscopic images of capsule staining with India ink of heterogeneous Kpn BJ1, a KL2 strain isolated from a liver abscess in France (left), and non-heterogeneous Kpn NTUH-K2044, a KL1 strain isolated from a liver abscess in Taiwan (right), after growth in LB medium. Upon contact with India ink, the capsule is revealed as a halo surrounding the individual cells, as it is not uptaken by the cell and contrasts with the dark background, allowing its direct visualization. Images were captured using a light microscope with oil immersion. The tube pictures show a representative image of a culture upon centrifugation through a Percoll gradient. Orange bar and blue triangle highlight the accumulation of cells in the different gradient layers. (B) Relative absorbance across the different layers of the Percoll gradient to identify heterogeneous and non-heterogeneous strains upon growth on LB medium. Non-capsulated mutants (∆*wcaJ*) were used as non-heterogeneous controls. Error bars indicate standard deviation. (C) Quantification of capsule production relative to the absorbance (OD_600_) for cells at the different gradient layers. Each gray dot corresponds to one individual experiment, and the blue dot and error bars represent the mean and the standard error, respectively. Kruskal-Wallis statistical analyses were performed using the function *stat_compare_means*() from the ggpubr package of R. Microscopy images across different layers are provided in Fig. S2. (D) Robust coefficient of variation of the FSC-A (Forward Scatter Area signal, a proxy for cell size) for *Kpn* strains (Cap+) and their respective non-capsulated mutants (Cap−) strain. A paired Kruskal-Wallis test was performed using the function *stat_compare_means*() from the *ggpubr* package of R. (E) Heterogeneity test of mutant strains producing less capsule (∆*rcsB*) and less hypermucoid (∆*rmpA*) upon growth on LB medium. Their respective wild types are shown in panel B). Error bars indicate the standard deviation of three independent replicates.

We observed that in some clonal populations of *Klebsiella pneumoniae,* cells produce various amounts of capsule (Fig. 1A). To measure the prevalence of this phenomenon across the *K. pneumoniae* phylogeny and separate bacterial populations based on capsule amount (34), we used discontinuous density gradients of Percoll. This methodology has previously allowed the successful screening of a transposon mutagenesis library and the identification of capsule regulators (26). Here, we increased the number of gradient layers from three (10%, 35%, and 50%) to five (10%, 20%, 30%, 40%, and 50%) to get a finer resolution of different capsulation levels (Fig. S1A). Upon slow centrifugation, hypocapsulated strains fall to the bottom through the gradient and are collected at the 50% fraction, as shown by non-capsulated controls (∆*wcaJ*, Fig. 1B), and highly capsulated strains are retained in the upper fraction (10%) (Fig. 1A and B). In heterogeneous populations, bacterial cells can be recovered across several fractions of the Percoll gradient, indicating the presence of hypercapsulated cells, hypocapsulated cells, and intermediate cells (Fig. 1A). By carefully removing each layer with the pipette, we could separate the different cells according to their capsule expression level. Different levels of capsule production across the layers of the gradient were confirmed in five heterogeneous *Klebsiella* strains by the traditional uronic acid method (Fig. 1C), and further supported by microscopy (Fig. S2).

To reproducibly quantify heterogeneity, we measured the absorbance of each gradient layer as a proxy of the number of cells. Alternatively, image analyses of photographs of the gradients after centrifugation can also be performed to evaluate heterogeneity (see Supplemental text, Fig. S3). The latter rather than binning the distribution of cells in five categories corresponding to each of the gradient layers, results in a continuum.

Then, we developed three different agnostic methods to identify whether a strain was heterogeneous, based on the absorbance readings from each layer. We first reasoned that heterogeneous strains would have an absorbance higher than 0 across several gradient layers. We consider that maximum heterogeneity occurs when there is a continuum in the degree of capsulation in the culture, that is, there is roughly the same amount of cells in the different binning categories, corresponding to the different gradient layers, thus approximately 20% of cells in each of the five different gradient layers. Using a set of 35 strains, we observed that when three different gradient layers contained at least 15% of the cells, the strains were heterogeneous (see Materials and Methods; Fig. S4A and B). Alternatively, using the same set of strains, we calculated the slope between the relative absorbance and the different Percoll gradient layers. Heterogeneous strains should have a slope near 0. Using a natural break in the slope distribution (Fig. S4C) and standard deviation of the slope, a slope range was determined and hereafter used as a cutoff for heterogeneity(Fig. S4C). Finally, we calculated the Shannon entropy index as a measure of heterogeneity (Fig. S4E). All three methods were similar, but the linear regression and the use of cutoff gave the same and more conservative results. Although heterogeneity was calculated by the three methods for all strains, figures hereafter report heterogeneity as inferred by the slope (Fig. S4F) (see Materials and Methods).

We then verified that the observed heterogeneity in capsule production is not due to differences in cell size. To do so, we performed flow cytometer experiments (Fig. 1D; Fig. S1B) and showed that there is no significant difference in variance in cell size between capsulated and non-capsulated strains, independently of whether they are heterogeneous or non-heterogeneous. We then checked whether heterogeneity is dependent on the amount of capsule production or hypermucoviscosity at the population level. We tested phenotypic heterogeneity in ∆*rcsB* mutants, known to produce less capsule than the wild type (26, 35), as well as ∆*rmpA* mutants, known to be less hypermucoviscous (36) (Fig. 1E). Despite some changes in individual fractions, the gradient layer (notably 10%) compared to the wild type (unpaired *t*-tests, *P* < 0.05), reducing capsule production (∆*rcsB*) or hypermucoviscosity (∆*rmpA*) does not result in a switch from a heterogeneous strain to a non-heterogeneous strain (and vice-versa).

Finally, to ensure that the observed heterogeneity is not due to the accumulation of fixed genetic mutations in our cultures, we grew a subset of heterogeneous strains and separated the different subpopulations in a gradient. We then reinoculated the top and lower layer of the gradient containing the hyper- and the hypo-capsulated strains in two different cultures. Once both subcultures reached the stationary phase, we confirmed that there were no significant differences between the initial and the two subcultures initiated from the different gradient layers, thus fully reproducing the heterogeneity of the original population (multi-way analysis of variance, *P* > 0.05 across cultures) (Fig. S1C).

Taken together, we developed a method to test agnostically phenotypic heterogeneity in capsule production in *Klebsiella pneumoniae*.

### Capsule phenotypic heterogeneity in *Klebsiella* spp. is widespread

**Fig 2:**
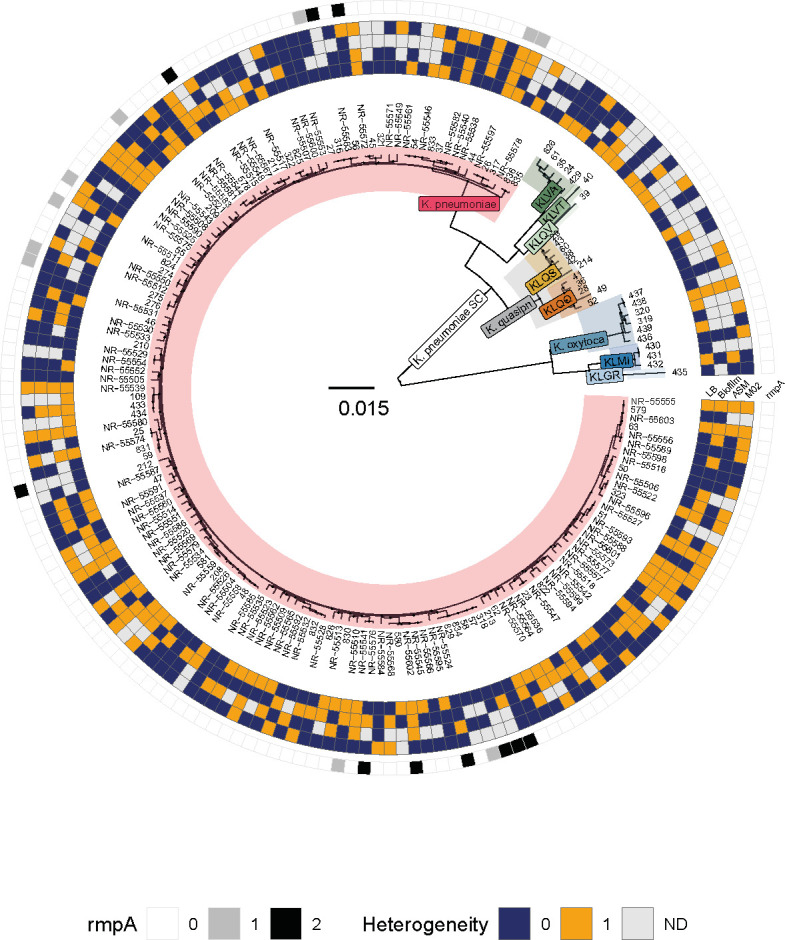
Phylogenetic tree based on the core genome of all *Klebsiella* strains used in this study. The tree was built using the PanACoTA pipeline (38) (see Materials and Methods). It can be directly accessed at https://microreact.org/project/mReVw42pW7YBsZNFuqKhuu-klebsiella-spp-tree. 0/1/2 indicates the number of *rmpA* alleles detected in each genome by Kleborate V2.0(39). Phenotypic heterogeneity (1, orange) and non-heterogeneity (0, blue) are indicated for each strain, grown in each environment. Light gray boxes represent those for which data were not produced. All individual data using the three methods are presented in Table S4. All tests were performed at least in triplicate.

To address how common capsule phenotypic heterogeneity is, we tested a panel of 159 *Klebsiella pneumoniae* strains, of which 100 belonged to the Multidrug-Resistant Organism Repository and Surveillance Network (MRSN) clinical diversity panel (37), and 59 from our collection and isolated from different sources. We observed that 44% of *K. pneumoniae* were heterogeneous in LB medium (Fig. 2). Moreover, we never observed an on/off bistability, where a subpopulation is hypercapsulated and another subpopulation is hypocapsulated, but rather a more subtle continuous distribution of the amount of capsule per cell (Fig. S5). We then tested other worrisome species belonging to the *Klebsiella* genus (Table S2). We found that at least one strain from each *Klebsiella* species exhibited heterogeneity (except for *Klebsiella grimontii,* for which only one strain was tested) (Fig. 2; Fig. S6). Overall, our data indicate that phenotypic heterogeneity in capsule production within a clonal population is a widespread trait across the *Klebsiella* genus.

### Heterogeneity depends on growth environment

**Fig 3:**
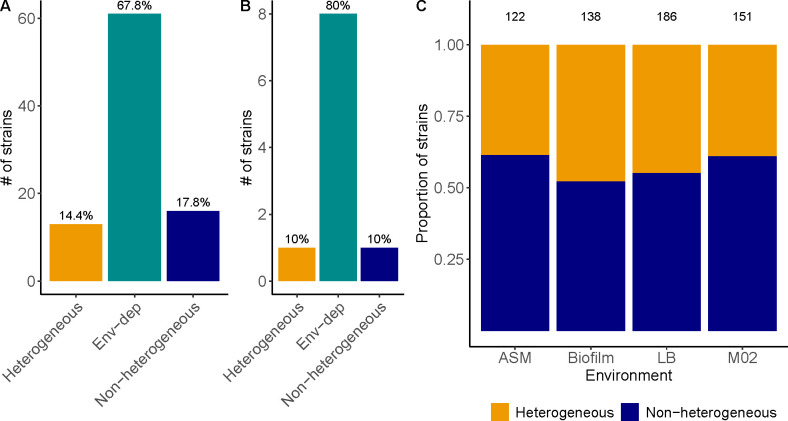
Heterogeneity depends on the environment. Heterogeneity was determined upon centrifugation of cultures across a Percoll gradient at least three times per strain and environment. Number of *K. pneumoniae* strains (A), other *Klebsiella* spp. (B) that are either heterogeneous or non-heterogeneous, regardless of the environment. Strains for which the phenotypic heterogeneity depends on the environment (Env-dep) are indicated in turquoise. Only strains that were tested in all four environments were included in the analyses. Numbers on top of bars indicate the percentage of strains. (C) Phenotypic heterogeneity of all *Klebsiella* spp. strains tested across environments. Numbers on top of bars indicate the number of strains tested.

Environmental conditions impact capsule production, including oxygen exposure (40). In most strains, capsule production increases in nutrient-poor media, where it provides a competitive advantage (33). We reasoned that if heterogeneity provided a fitness advantage when the capsule imposed a high cost, it could become homogeneous under different environmental challenges. Specifically, we hypothesized that (i) more strains would be heterogeneous when grown as biofilms, as these matrix-encased communities are environments in which heterogeneity is fueled (41), and (ii) in nutrient-poor environments, where more capsule is produced (33), strains would be less heterogeneous due to a larger proportion of hypercapsulated cells. To test this, we measured capsule heterogeneity in a large random subset of strains in nutrient-rich artificial sputum (ASM), nutrient-poor media (M02), and colony biofilms grown on agar pads (Fig. 3).

Our results reveal that 68% of *K. pneumoniae* strains and 80% of other *Klebsiella* strains showed phenotypic heterogeneity in at least one out of the four environments tested (Fig. 3A and B, and Fig S6). We showed that, overall, a larger proportion of *Klebsiella* spp. strains were heterogeneous in biofilm (45% vs 51%), but this was not statistically significant (X^2^ = 0.38, *P* = 0.53) (Fig. 3C). We also observed changes in heterogeneity depending on the nutrients available in the environment (Fig. S7 and S8). Only 15% of the strains maintained their phenotype (heterogeneous or homogeneous) across all environments (Fig. S7A). In both nutrient-rich media (LB and ASM, Fig. S7A), most strains showed the same phenotype but differed in nutrient-poor media (Fig. S7 and S8). However, the direction of change was strain-dependent and independent of the environment (X^2^ = 1.9, *P* = 0.38) (Fig. S7 and S8).

We then tested whether the previously described increase in capsule production in nutrient-poor media is due to a shift of cells from hypocapsulated states to more hypercapsulated states. If changes in population-level capsule production were not dependent on phenotypic heterogeneity, we would expect to observe no difference in the proportion of cells in each gradient layer between nutrient-rich and nutrient-poor media, as all cells would increase in the same degree of capsule production (Fig. S9A). However, we do observe that in populations that produce more capsules in nutrient-poor media, a higher proportion of cells accumulate at the upper gradient layers, whereas in nutrient-rich media, more cells tend to accumulate in the lower levels of the gradient (Fig. S9B). The opposite is true for those strains that produce less (Fig. S9C). As expected, strains producing similar amounts of capsule in both environments display similar patterns of heterogeneity (Fig. S9C). This suggests that changes in population-level capsule production are not only a result of generic changes in gene expression across environments, but also rely on phenotypic heterogeneity and the shift of hypocapsulated cells into more capsulated cells.

Taken together, although there is no trend across environments and heterogeneity is both strain- and environment-dependent, most strains are heterogeneous in at least one environment.

### Capsule locus shapes phenotypic heterogeneity

**Fig 4:**
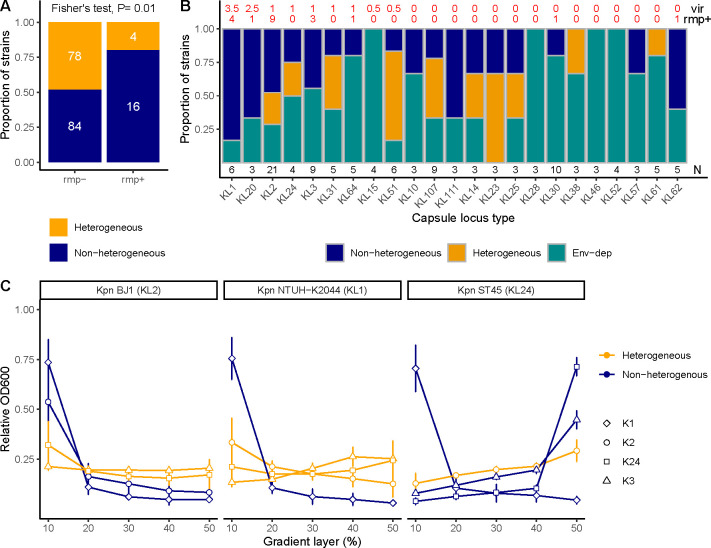
Phenotypic heterogeneity is encoded, at least partly, by the capsule locus. (A) Proportion of heterogeneous strains depending on the presence of the *rmpA* gene, as determined by Kleborate V2.0(39). Both *rmpA* and *rmpA2*, whether chromosomal or plasmid-encoded, were taken into account. In white, the number of strains belonging to each category is indicated. Excluding truncated *rmp* does not qualitatively alter the results. Fisher’s Exact test and X^2^ both result in *P* < 0.05. (B) Phenotypic heterogeneity capsule locus types (KL) were identified using Kleborate V2.0(39). Numbers below the bars indicate the sample size (N). Serotypes are ordered according to their median virulence score (vir). Indicated below (rmp+) are the number of strains from each serotype encoding *rmp* alleles. (C) Phenotypic heterogeneity in capsule production of cultures of three different genetic backgrounds expressing different capsule serotypes was determined upon centrifugation across a Percoll gradient. Control experiments are shown in Fig. S10, and microscopy images are presented in Fig. S11. Error bars correspond to standard deviations from the mean (at least three independent biological replicates).

Changes in patterns of phenotypic heterogeneity of capsule production in response to the environment strongly suggest that this is at least partially genetically encoded. The lack of phylogenetic inertia in the distibution of heterogeneity across the strains could reflect that the loci involved could be explained by extensive horizontal gene transfer. The capsule locus and the major regulator *rmp*, mostly transferred in a plasmid (27) and seldom present in the chromosome, are subject to extensive genetic transfer. We first tested whether the presence of *rmp*, common in hypervirulent strains, correlated with phenotypic heterogeneity. Our data reveal that capsule heterogeneity is associated with the absence of the *rmpA* and *rmpA2* alleles (Fig. 4A). We then tested whether heterogeneity negatively correlated with virulence score based on the presence of several virulence factors, as calculated by Kleborate V2.0(39). This suggested that heterogeneity is more common in classical *K. pneumoniae* strains than in hypervirulent strains (i.e., with a lower virulence score [GLM, *P* = 0.056], as predicted by genetic content [Table S3]).

Our results also suggest that certain capsule locus types (CLTs—as predicted bioinformatically), like K1 and K2, associated with hypervirulence, would be less likely to be heterogeneous. Indeed, K1, alongside K20, KL111, and K62, were the only CLTs for which 50% of the strains were non-heterogeneous across all environments (Fig. 4B).

We then tested whether specific serotypes could result in capsule heterogeneity. To do so, we leveraged a collection of three different genetic backgrounds (Kpn BJ1 -KL2-, Kpn NTUH K2044 -KL1-, and Kpn ST45 -KL24-) expressing one of four different capsule serotypes (K1, K2, K3, and K24) (12). Control experiments in which the native serotype was reinserted in the non-capsulated mutant did not result in changes in heterogeneity between the wild-type strain and the complemented strain (Fig. S10). We observed that all strains expressing K1 were non-heterogeneous (Fig. 4C). This is in line with the abovementioned finding that most KL1 CLT strains are non-heterogeneous (Fig. S7; Fig. 4B). This is so even in the absence of *rmp* (Kpn ST45). For the three other serotypes, we did not observe a common pattern of phenotypic heterogeneity across the different genetic backgrounds. In *Kpn* ST45, capsule production is mostly non-heterogeneous, except when the K2 capsule locus is expressed. The exchange of K24 into strain NTUH K2044 and BJ1 resulted in a gain of heterogeneity (Fig. 4C; Fig. S11), but K24 in its native genetic background (ST45) is non-heterogeneous. These results show that heterogeneity is partially determined by the capsule type, i.e., partially horizontally inherited with the capsule.

### Phenotypic heterogeneity limits capsule cost and shapes phage adsorption

**Fig 5:**
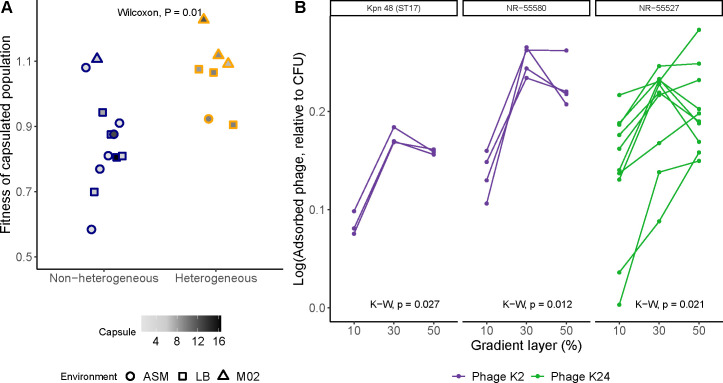
Heterogeneity increases fitness and phage adsorption. (A) Fitness of heterogeneous and non-heterogeneous strains. Capsulated strains were competed against their respective non-capsulated strains in an initial 1:1 ratio. The fitness of the capsulated strain is displayed. Each dot represents the mean of at least three independent biological replicates. Error bars are not shown for visibility purposes. Different shapes indicate different growth conditions. The intensity of the gray inside the dots indicates the amount of capsule produced in the growth condition. Considering only competitions in LB medium does not alter the result (Fig. S12). Data from competitions were, in part, originally published in Buffet et al. (33). (B) Cells from different gradient layers were put in contact with phage for 10 min (MOI ~1). Cell-bound phages were discarded, and free, non-adsorbed phage particles were then quantified and expressed relative to the total CFUs. Data is displayed as log_10_. Each line represents an independent biological replicate. Phage phK2 and phage phK24 (12) were used to infect the KL2 and KL24 strains, respectively. Statistics correspond to a Kruskal-Wallis (K-W) test.

The prevalence of capsule heterogeneity raised the question of whether it provides a specific fitness advantage. We thus tested whether heterogeneous strains were, on average, fitter than non-heterogeneous strains. To circumvent differences across genotypes, we competed capsulated wild-type strains against their respective non-capsulated mutants and compared the fitness of each capsulated strain. We show that on average, heterogeneous populations are more fit than non-heterogeneous strains (Fig. 5A). This trend persists even when focusing only on competitions performed in nutrient-rich LB medium, where capsule production is costly (33) (Fig. S12). Notably, this fitness advantage occurs despite heterogeneous strains producing more capsule overall than non-heterogeneous ones (x̄ = 9.8 for heterogeneous and x̄ = 6.1 for non-heterogeneous, as measured at the population-level by the uronic acid method, *t*-test, *P* = 0.04; Fig. 5A). In non-heterogeneous populations, there is no correlation between capsule production and fitness (Spearman, *P* = 0.78, rho = 0.11). In nutrient-poor media, where the capsule is known to increase fitness against non-capsulated strains (33), heterogeneity does not further increase such an advantage (Wilcoxon test, *P* = 0.4).

Capsule heterogeneity could also increase bacterial survival, and most notably during phage predation, as the capsule is required by most for adsorption and subsequent infection (12, 23, 24). We thus tested whether phage could adsorb differently to cells producing different amounts of capsule (Fig. 5A). Our results show that subpopulations with more capsule absorbed less phage, whereas populations with intermediate levels of capsule adsorbed more phage. Further reduction of capsule expression did not consistently result in more phage adsorption (Fig. 5B).

Overall, the heterogeneity in the expression of the capsule dampens the fitness burden of capsule production and could allow a certain subpopulation to escape from phage predation by limiting adsorption.

### Capsule phenotypic heterogeneity is also observed among other capsulated bacteria

Many facultative pathogens are capsulated and known to display some degree of heterogeneity. We thus tested some strains from closely related enterobacteria, namely, three *E. coli* strains encoding two different capsule types: IAI1 and 55898 with group 1 capsules and *E. coli* CFT073 with a group 2 capsule. All *E. coli* cells were collected at the bottom layer, indicative of hypocapsulation (Fig. S13A). However, it could also reflect a limit of detection of the Percoll gradient. Indeed, despite being encapsulated, the *E. coli* capsule volume may not be large enough to be retained by the gradient. To test this, we took advantage of an *E. coli* strain encoding a capsule that had been transferred horizontally from *Klebsiella variicola* (42). This strain produced a large capsule visible with an India ink staining, whereas the capsulated CFT073 is not visually different from its non-capsulated ∆*kpsM* mutant (Fig. S13B and C). *E. coli* Ec300, albeit non-heterogeneous, was retained in the Percoll column (Fig. S13B), revealing that the use of Percoll gradients is relevant for heavily capsulated species, but has a lower limit of detection.

**Fig 6:**
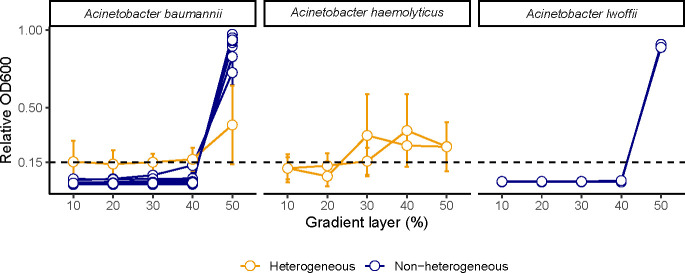
Phenotypic heterogeneity in capsule production is also observed in *Acinetobacter* spp. Each line corresponds to a different strain (see Table S2 for strain details). Error bars correspond to standard deviations (three independent biological replicates).

Given this limitation, we then expanded our search to test whether this phenomenon could be common to other heavily capsulated bacteria. We tested several species of *Acinetobacter,* including *A. baumannii* (*N* = 13)*, A. lwoffi* (*N* = 2), and *A. haemolyticus* (*N* = 2) (Table S2). The use of all three heterogeneity methods independently identified both *A. haemolyticus* and one *A. baumannii* (strain ATCC 17978) strain as heterogeneous (Fig. 6). These results were also confirmed by the determination of the Shannon diversity index (Fig. S13D). Overall, we show that phenotypic heterogeneity is not restricted to *Klebsiella* species but could be a common trait across capsulated species.

## DISCUSSION

We developed a solid, reproducible, agnostic, and unbiased methodology to determine heterogeneity in capsule production using density-based separation by gradient centrifugation. We show that it is prevalent across *K. pneumoniae* and across other species of the *Klebsiella* and *Acinetobacter* genera. Phenotypic heterogeneity could play a major role in shaping the alternation of the bacterial life cycle between the environment and pathogenesis, as it confers a small population of bacteria with useful and reversible functionalities in a short period of time. Indeed, it could provide clues as to *Klebsiella* persistence within the host. The changes between commensalism and pathogenesis are often complex and not well studied. There is contradictory evidence on whether the capsule is required for *Klebsiella* gut colonization (43–45), yet it is an advantage for bloodstream infections (43, 46, 47). The existence of phenotypic heterogeneity would provide a significant advantage during commensalism by providing for transient benefits, without compromising the capacity to generate bloodstream infection. This is opposed to genetic changes, which could hamper future opportunistic *Klebsiella* infections, as mutations are, in most cases, difficult to revert (48). Furthermore, the benefit of hypocapsulated cells could be twofold. First, it minimizes capsule cost in environments in which it does not provide a large advantage. And second, since less-capsulated bacteria are prone to conjugating better, it provides increased opportunities to acquire antibiotic resistance and virulence genes (12).

We reasoned that the phenotypic heterogeneity could be due to a mutational event, to noise in gene expression (49), the result of cells being in different metabolic phases, or it could be controlled epigenetically (1). First, we demonstrate that the regrowth of either hypo- and hyper-capsulated cells fully recovers the original heterogeneity and in the same proportions within a few generations (Fig. S1C). One possibility could be that bacteria actively shed off the capsule, an analogous process of cell wall shedding in the presence of phage in other bacteria (50). Alternatively, heterogeneity could be determined by DNA methylation patterns, which can be efficiently diluted or reset in a couple of generations. Although at this point, the mechanisms remain purely speculative, it strongly indicates it is not due to a mutation. Second, we show that heterogeneity is highly reproducible, present in some strains, but not all, subject to changes depending on the environment, and relies on specific epistatic interactions between capsule and genetic background. We thus exclude that it is due to the fact that cells are in different growth phases. We thus postulate that phenotypic heterogeneity may be epigenetic. However, it still relies on the existence of specific sequences both in the capsule locus and the genetic background, or epistatic interactions between them. Indeed, insertion of a heterogeneous capsule in a specific genetic background did not always result in heterogeneity in other backgrounds.

It is, however, noteworthy the dichotomy observed between the non-hypervirulent, or classical, *K. pneumoniae,* which are in most part heterogeneous, and those encoding *rmpA* (regulator of mucoid phenotype) and predicted to be hypervirulent strains, which are non-heterogeneous and locked in hypercapsulation. A recent study reported that insertion or deletion of one T residue in a simple sequence repeat (SSR) in the promoter of *rmpA* resulted in altered lethality, cell adhesion, and colonization (31). It was later shown that this also had a large impact on mucoidy and capsule production (32). It is tempting to speculate that this mechanism is analogous to the phase variation in capsule expression in *N. meningitidis* (13)*,* also regulated by an SSR. In combination, these data suggest that both classical and hypervirulent *K. pneumoniae* would be able to rapidly adapt by modulating capsule production.

Previous studies have suggested that resistance to phages, including in *K. pneumoniae* (51–53), could be transient and non-genetic, a phenomenon known as phenotypic resistance. This could be explained by heterogeneity in the expression of the phage receptor. We show that cells in different gradient layers adsorb phage differently (a prerequisite for infection). Interestingly, hypercapsulated cells adsorb less than other less capsulated cells. This is in line with studies suggesting that although the capsule is necessary for primary (reversible) attachment, it is not the final receptor (54). Excessive capsule production would mask and reduce accessibility to the final receptor. Similarly, it could be that more phage (with more capsule depolymerases) is required to degrade all the capsule in hypercapsulated strains. Interestingly, our data also show that the less capsulated cells (layer 50%) do not adsorb more than the intermediate capsulated cells. It could be that there is an accumulation of some non-capsulated mutant cells, which cannot be differentiated by the Percoll gradient from hypocapsulated cells, and which are unable to adsorb phage (12, 53). Capsule heterogeneity could thus constitute a strategy to better cope with phage pressure and allow a subpopulation to escape infection, in a process analogous to persistence during antibiotic exposure. Our data suggest that there is an intermediate capsule optimum for phage adsorption, which would require some capsule for recognition but still allows access to the final receptor. In other species, like *Bacteroides fragilis*, a gut commensal expressing several capsule types, heterogeneity at the single-cell level has been recently revealed. This results in different combinations of capsule types, alongside differential expression of restriction-modification systems (a well-known intracellular defense system) and fimbrial genes, which together serve as a mechanism of phage evasion, by which a subpopulation totally escapes killing (55). Phenotypic resistance to phages is not constricted to capsule heterogeneity, but is also known in other surface structures. For example, a bistable switch of the O-antigen results in resistance to phages in *Salmonella* Typhimurium (56).

We show that heterogeneity can vary across environments. Counterintuitively, heterogeneity was not exacerbated when cells grew within biofilms. This was expected given the intrinsic environmental heterogeneity due to nutrient, waste, and oxygen gradients, which oftentimes results in physiological heterogeneity (57). However, the strains used in this study were collected from diverse environments with varying degrees of spatial structure and thus may be already locally adapted. As a result, their behavior and their phenotypic heterogeneity might differ upon exposure to other environmental conditions. More importantly, we showed that capsule production at the population level could not only be altered by direct transcriptional changes, but can also be impacted by changes in the heterogeneity and, notably, the proportion of hyper- and hypo-capsulated cells within a population. This may be an optimal way to adapt to environments in which the capsule is more costly, without a transcriptional rewiring of core metabolic functions that are intricately related to capsule production (25, 58). We show that heterogeneity limits the cost of capsule (Fig. 5A), especially in strains where more capsule is produced. This suggests a stabilization of capsule heterogeneity toward an optimum and could be indicative that it is an adaptive trait.

The method to quantify heterogeneity presents some limitations. It is not applicable to capsules whose total volume is too small, even those from closely related genera. Indeed, the capsule thickness of *E. coli* is estimated at around 10 nm, whereas classical *K. pneumoniae* strains have capsules measuring approximately 160 nm, and hypercapsulated cells can reach 300–400 nm (59, 60). For example, it is known that group 2 capsules present in uropathogenic *E. coli* are phenotypically heterogeneous (16). However, our method does not allow us to demonstrate that this was so in uropathogenic CFT073. Whereas this limitation does not challenge the conclusions of our analyses, it underscores a caveat in applying the method to a broader range of bacterial species. Future experiments would be needed to test whether further dilutions of Percoll, below 50%, would provide robust gradients to test smaller or lightly-capsulated bacteria at a much finer scale. Additionally, here we considered strains to be either heterogeneous or non-heterogeneous, but all three methods showed that distributions are rarely binomial, and some strains are more heterogeneous than others (Fig. S4A and C). Future analyses could focus on the degree of heterogeneity, as both the entropy measurement and slope of linear regression provide an opportunity to quantify heterogeneity. Finally, another improvement to the proposed methodology could be the use of image analyses, both of microscopy imaging and tube photographs, for a more rapid screening of phenotypic heterogeneity (Text S1).

Given the importance of the capsule in the bacterial life cycle, our results open many different research venues. Upon treatment with antimicrobial peptides, hypocapsulated cells could display higher survival rates, as the capsule increases cell sensitivity (35). Also, capsule heterogeneity may have important implications in bacterial evasion from the innate immune system and could explain the success of *K. pneumoniae* in the transition from healthy colonization to systemic infection. Aside from the described benefits upon phage predation, heterogeneity can have important repercussions in bacterial evolution as a result of the specific interactions with mobile genetic elements (12). Indeed, heterogeneity could fuel lineage divergence, by which a subset of cells is more exposed to phages and another subset is more exposed to conjugative plasmids. These mobile genetic elements carry different bacterial genes (61) and could thus lead to different evolutionary trajectories. Finally, capsule heterogeneity should also be taken into account in translational research, as the capsule is a major target of preventive approaches (vaccines) and treatment (phage therapy) against infections.

## MATERIALS AND METHODS

### Bacterial strains

#### Wild-type strains

The strains used in this study, as well as their genomic annotations and accession numbers, are described in Table S1. *Klebsiella pneumoniae* strains came from our laboratory collection and from the MRSN collection (37). The MRSN strains were mostly collected in a clinical setting and represents a diverse set of *K. pneumoniae* covering many multi-locus sequence types (MLST), with quality genomic data and curated metadata available.

Strains in our laboratory collection were selected based on MLST data, their origin of isolation, and were representative of the phylogenetic and clonal diversity of the *K. pneumoniae* species complex. Other strains, including all of *Klebsiella* spp., *Acinetobacter* spp., and *Escherichia coli,* are described in Table S2. Capsule locus types predicted by bioinformatics are noted as KL (i.e., KL1, KL2), whereas serologically determined capsules are referred to as K (i.e., K1, K2).

#### Capsule mutants

All mutants used in this study are described in Table S5. (i) *Non-capsulated mutants*. Mutants used as controls and for the competitions were generated isogenically by double recombination events resulting in allele replacement. These mutants have an in-frame deletion of either *wcaJ* (the first gene in the biosynthetic pathway of the capsule) or *wza* (the outer membrane porin) and were published in Buffet et al. (33). (ii*) rmpA mutants*. As for non-capsulated strains, mutants were generated by double recombination events resulting in allele replacement, as described in reference 33. Mutants were verified by Sanger sequencing and by the reduction of hypermucoviscosity. (iii*) Capsule exchanges*. The strains with exchanged capsules were described previously (12). Briefly, capsule deletion was performed by recombination at the homologous regions (*galF* and *ugd*), replacing the capsule locus with a cassette containing a kanamycin resistance gene, one I-Scel cut site, and two FRT sites. The resistance gene was then excised by the flippase acting on the FRT sites. In parallel, the K1, K2, K3, and K24 capsules were cloned using a pKAPTURE cassette, which circularizes around the capsule locus via recombination (around *galF* and *ugd*). Finally, the cassette containing each capsule locus was transformed into each isogenic non-capsulated genetic background (Kpn BJ1, Kpn NTUH-K2044, and Kpn ST45). The cassette was then integrated into the deletion mutant by recombination between the two homolog regions, facilitated by I-SceI endonuclease cuts. This method allows integration of the desired serotype into any genetic background. Each strain only encodes one capsule serotype, and capsule production was verified (12). Control experiments compare the wild-type strain and a complemented strain encoding the native capsule loci.

#### Cultures

To initiate all experiments, single bacterial colonies were inoculated in 4 mL of fresh LB and incubated overnight at 37°C under shaking conditions (250 rpm), unless stated otherwise.

### Capsule phenotypic heterogeneity

#### India ink staining and visualization

Ten microliters of overnight bacterial culture was resuspended in 5 µL of India ink on the slide. The coverslip was carefully placed on the dye-bacteria mixture. Using a paper towel, pressure was carefully applied to press down on the coverslip until the slide appeared as a photographic negative. Cell morphologies were imaged using a Zeiss Axioplan 2 microscope, with ×1,000 magnification, equipped with an Axiocam 503 mono camera (Carl Zeiss, Germany). Images were acquired using the ZEN lite software (v. 3.6). ImageJ software (v. 2.16.0) was used to adjust contrast and add the scale bar.

#### Percoll gradients

Percoll solutions of 10%, 20%, 30%, 40%, and 50% were prepared freshly prior to the experiment by diluting Percoll in PBS 1×. To generate the gradient, 1 mL of each Percoll solution was carefully layered in 14 mL tubes, starting from the highest to the lowest concentration.

#### Preparation of bacterial cells

##### (i) Liquid cultures

For quantification of heterogeneity in LB and ASM (nutrient-rich media), overnight cultures were centrifuged at 4,500 rpm for 10 min, the supernatant discarded, and cells were resuspended in 1 mL of PBS. For cells growing in nutrient-poor media (M02 - M63B1 supplemented with 0.2 % glucose), an overday culture in LB was allowed to grow and used to inoculate, in a 1:100 ratio, an overnight culture of M02. Six hundred microliters of the resuspended cells were added on top of the Percoll gradient.

##### (ii) Biofilm cultures

Each overnight culture was diluted 1:100 in fresh LB, and 50 uL of this dilution was deposited onto two wells of a 24-well plate containing 1.5 mL of LB agar each. Biofilms were grown at 37°C statically for 24 h. To recover the cells, 300 µL of 1× PBS was added to each well, and the plate was shaken for 15 min. Two technical replicates for each strain were combined, and 600 µL of the resuspended cells were added on top of the Percoll gradient.

### Quantification of heterogeneity

Once the resuspended bacterial culture was added to the Percoll gradient, it was centrifuged at 2,100 rpm for 30 min. Cells retained at the top correspond to the largely capsulated, whereas cells at the bottom correspond to the hypocapsulated or non-capsulated. When a large cell pellet was observed at the bottom, the biological replicate was discarded, as it indicates that non-capsulated mutants rapidly accumulated during growth in rich media.

#### (i) Absorbance method

The different gradient layers were separated by carefully pipetting 1 mL at a time, and the absorbance (OD_600_ ) of each phase was measured with a spectrophotometer using 1.6 mL cuvettes. To homogenize data across strains and replicates, for each sample, the OD_600_ of each layer was divided by the sum of all OD_600_ as a measure of the proportion of cells in each layer.

#### (ii) Imaging method

Photographs of each tube upon centrifugation through the Percoll gradients were taken and analyzed using ImageJ software (62). Briefly, the images were converted to grayscale format (8-bit). Then, a line was drawn along the Percoll gradient using the Straight tool, a Plot Profile was generated, and the Gray Values were extracted for plotting.

### Determination of heterogeneity

Three different methods to determine automatically and agnostically heterogeneity were developed using 35 different *Klebsiella* strains.

#### (i) Cell proportion

We estimated that to consider a strain heterogeneous, a certain proportion of cells had to be present in three or more gradient layers. Taking into account that the maximum proportion for a totally heterogeneous strain would be 0.2, we performed the distribution of the number of layers that had at least a proportion of cells above 0.1, 0.15, and 0.2. This revealed that 0.2 was very restrictive, but 0.1 and 0.15 showed distributions close to bimodal (Fig. S4A). We compared both, and out of the 35 strains, 22 and 16 strains were either heterogeneous or not depending of the cutoff used (Fig. S4B). Given that the distribution of the six strains for which heterogeneity was dependent on the cutoff, and in order to be conservative, we settled for a cutoff of 0.15.

#### (ii) Linear regression

We considered that a slope near zero would be indicative of heterogeneity, whereas large positive or negative slopes would reflect populations homogeneously hypercapsulated (such as NTUH-K2044, hypervirulent, KL1) or populations hypocapsulated (Kpn NR-55507, MDR KL38). We used a linear regression to calculate the slope of the relative proportion of cells across the different gradient layers and established a cutoff between 0.008 and −0.006, within which *Klebsiella* strains are considered heterogeneous (Fig. S4C). The upper cutoff of 0.008 is well defined, as there is no overlap in the slope standard deviations of the slopes (Fig. S4D). For the lower cutoff, no natural cutoff was observed, and it was established at −0.006 as being the most parsimonious. Using these two cutoffs, for the subset of strains used, there are no differences between the two methods.

#### (iii) Shannon entropy

Entropy is a classical method to measure heterogeneity in a sample. To determine whether the distribution of cells across the gradient layers is heterogeneous, we used the diversity() function, index=“shannon,” included in the vegan package for R (https://github.com/vegandevs/vegan). This measure was extremely sensitive to very minor changes, due to experimental variations (Fig. S4E), as shown by frequent severe outliers. We thus considered the median, rather than the mean, which was more representative of the heterogeneity of the sample.

We then overlapped all results obtained from each biological replicate across all environments tested with the three methods (Fig. S4F). We decided to perform all analyses with *Klebsiella* strains using the slope from the linear regression model, as it is the most conservative method. Indeed, all strains but one that were identified as heterogeneous by this method were also identified as heterogeneous by at least one of the two other methods (Fig. S4F). For *Klebsiella* spp., all data for each strain using each method are presented in Table S4.

The use of either methodology for *E. coli* and *Acinetobacter* species did not alter the results (Fig. S13D).

Finally, we considered that heterogeneity in capsule production is environment-dependent, if a strain displays a different behavior in at least one out of the four growth conditions.

### Flow cytometry analyses

Flow cytometry analyses were performed to test differences in cell size. Precultures of each strain were grown overday in LB and then diluted to 1:100 in M63B1 minimal medium supplemented with 0.2% glucose (M02) for overnight culture. The latter were diluted to 1:200 in cold PBS 1×. Cell size was then acquired by fluorescence-activated cell sorting (FACS) analysis using a CytoFlex S. At least 4,000 events per strain were acquired. FACS results were analyzed using FlowJo v.10. Experiments were repeated three times.

### Capsule quantification

To quantify the amount of capsule per cell in each gradient layer, overnight cultures in LB medium were transferred to a Percoll gradient, as described above. After slow centrifugation, the layers were separated carefully with a pipette, and their OD_600_ was measured. Then, 500 µL of the gradient layers was transferred to an Eppendorf tube with Zwittergent to proceed to the capsule extraction as previously described (63). Then, cell-bound surface polysaccharides were quantified using the traditional uronic acid method (64). To determine the capsule amount, the uronic acid concentration of each sample was determined from a standard curve of glucuronic acid. To normalize for the number of cells, the concentration was then divided by the OD_600_ of each gradient layer. Capsule quantifications of bulk populations used to control fitness experiments or changes in heterogeneity with respect to capsule production were obtained from Buffet et al. (33).

### Genomic analyses and phylogenetic tree

To compute the phylogenetic tree, we used the PanACoTA v1.4.0 pipeline (38). First, we calculated the pangenome using the module pangenome. Gene families were built with MMseqs v14-7e284, with an identity and bi-directional coverage threshold of 80%. This analysis resulted in 35,928 gene families among the 187 genomes (Tables S1 and S2). The core genome was then computed and resulted in 450 gene families, with each genome having exactly 187 members, from the 187 different genomes. We then aligned each protein family of the core genome individually with the align module, which used MAFFT --auto (65). These alignments were concatenated to produce a large alignment matrix with 77,156 parsimony-informative sites over a total alignment of 417,069 bp. We then use this alignment to make the phylogenetic inference using IQ-TREE (version 2.2.2.2), using the best-fit model (GTR). We rooted the phylogenetic tree at the midpoint.root() function from the Phangorn R package.

Heterogeneity information was mapped onto the core-genome phylogeny using the package ggtree in RStudio, running R version 4.3.1. The tree can be directly accessed at https://microreact.org/project/mReVw42pW7YBsZNFuqKhuu-klebsiella-spp-tree. We used Kaptive v2.0.0 with default options and the “K locus primary reference” to identify the capsule locus types (K-locus) of the strains (39).

### Phage adsorption experiments

Phage lysates were acquired from several laboratories and streaked for single plaques on lawns of the wild-type strains BJ1 (phK2) and ST45 (phK24) as described previously (12). To test phage adsorption to cells expressing different amounts of capsule, we separated the different subpopulations through a Percoll gradient as described above. Layers corresponding to 10%, 30%, and 50% Percoll concentrations were selected and diluted in 1× PBS to adjust to the phase with the lowest optical density (ca. OD_600_ = 0.4, ~10^8^ CFU/mL). To evaluate the absorption capacity of the phage, a phage stock solution was diluted to 10^8^ PFU/mL and mixed in a 1:1 ratio (MOI = 1) with cells from each Percoll gradient layer. The mix was incubated at 37°C for 10 min. Tubes were then placed on ice and transferred to a pre-chilled centrifuge at 4°C. After centrifugation at 10,000 × *g* for 5 min, serial dilutions of the supernatant were spotted on bacterial lawns of the phage host for plaque (PFU) counting. Quantification of non-adsorbed phage was evaluated as previously described (53). Kpn BJ1 was used as prey for phage K2, whereas NR-55527 was used as prey for phage K24. Phages were amplified several times independently to ensure no batch effects.

### Competitions

Fitness values of non-heterogeneous and heterogeneous strains in competition with their respective non-capsulated strains were taken from, or performed as, in Buffet et al. (33). Briefly, non-capsulated mutants and their respective wild types were grown overnight in LB. They were then mixed in a 1:1 proportion. The co-culture was then diluted 1:100 in 4 mL of the different growth media (i.e., LB, ASM, and M02). A sample was taken and used for serial dilution and CFU counting (T_0_). After 24 h of growth (T_24_), each coculture was serially diluted and plated. Capsulated and non-capsulated colonies are differentiated visually and counted separately. The competitive index of capsulated strains was calculated by dividing the ratio of CFU at T_24_ over T_0_.

### Statistics

All experiments were performed at least in triplicate, including heterogeneity tests for all strains in each of the environments. Statistics were performed with R v4.3.1.

## Data Availability

Raw data are available at the following link: 10.6084/m9.figshare.29656499. Phylogenetic tree and data can be directly accessed in https://microreact.org/project/mReVw42pW7YBsZNFuqKhuu-klebsiella-spp-tree.

## SUPPLEMENTAL MATERIAL

Supplemental Figures, Part 1 mbio.01807-25-s0001.docx Figures S1 and S2.

Supplemental Figures, Part 2 mbio.01807-25-s0002.docx Figures S3 to S6.

Supplemental Figures, Part 3 mbio.01807-25-s0003.docx Figures S7 and S8.

Supplemental Figures, Part 4 mbio.01807-25-s0004.docx Figures S9 to S13.

Supplemental Tables mbio.01807-25-s0005.xlsx Tables S1, S2, S4, and S5 and data for Fig. 4A.

Supplemental Material mbio.01807-25-s0006.docx Supplemental text, Table S3, and supplemental table captions.

ASM does not own the copyrights to Supplemental Material that may be linked to, or accessed through, an article. The authors have granted ASM a non-exclusive, world-wide license to publish the Supplemental Material files. Please contact the corresponding author directly for reuse.
