# Supplementary material for: Phenotypic heterogeneity of capsule production across opportunistic pathogens
Source: mBio. 2025 Sep 4;16(10):e01807-25. doi: 10.1128/mbio.01807-25 (PMC12505892; doi:10.1128/mbio.01807-25)
Supplement: Supplemental Figures, Part 1 — Figures S1 and S2. [file mbio.01807-25-s0001.docx]

# SUPPLEMENTARY MATERIAL for

## Bet hedging of capsule production across opportunistic pathogens

Amandine Nucci^1#^, Julie Le Bris^1,2#^, Sara Diaz Diaz^3#^, Lilibeth Torres-Elizalde^3^, Eduardo P.C. Rocha^1^ and Olaya Rendueles*^1,3^

^1^Institut Pasteur, Université Paris Cité, CNRS UMR3525, Microbial Evolutionary Genomics, Paris 75015, France.

^2^Sorbonne Université, Collège Doctoral, École Doctorale Complexité du Vivant, 75005 Paris, France

^3^Laboratoire de Microbiologie et Génétique Moléculaires (LMGM), CNRS UMR5100, Centre de Biologie Intégrative (CBI), Université de Toulouse, CNRS, Université de Toulouse, Toulouse, France

# equal contribution

*Corresponding author, olaya.rendueles-garcia@utoulouse.fr

## SUPPLEMENTARY FIGURES


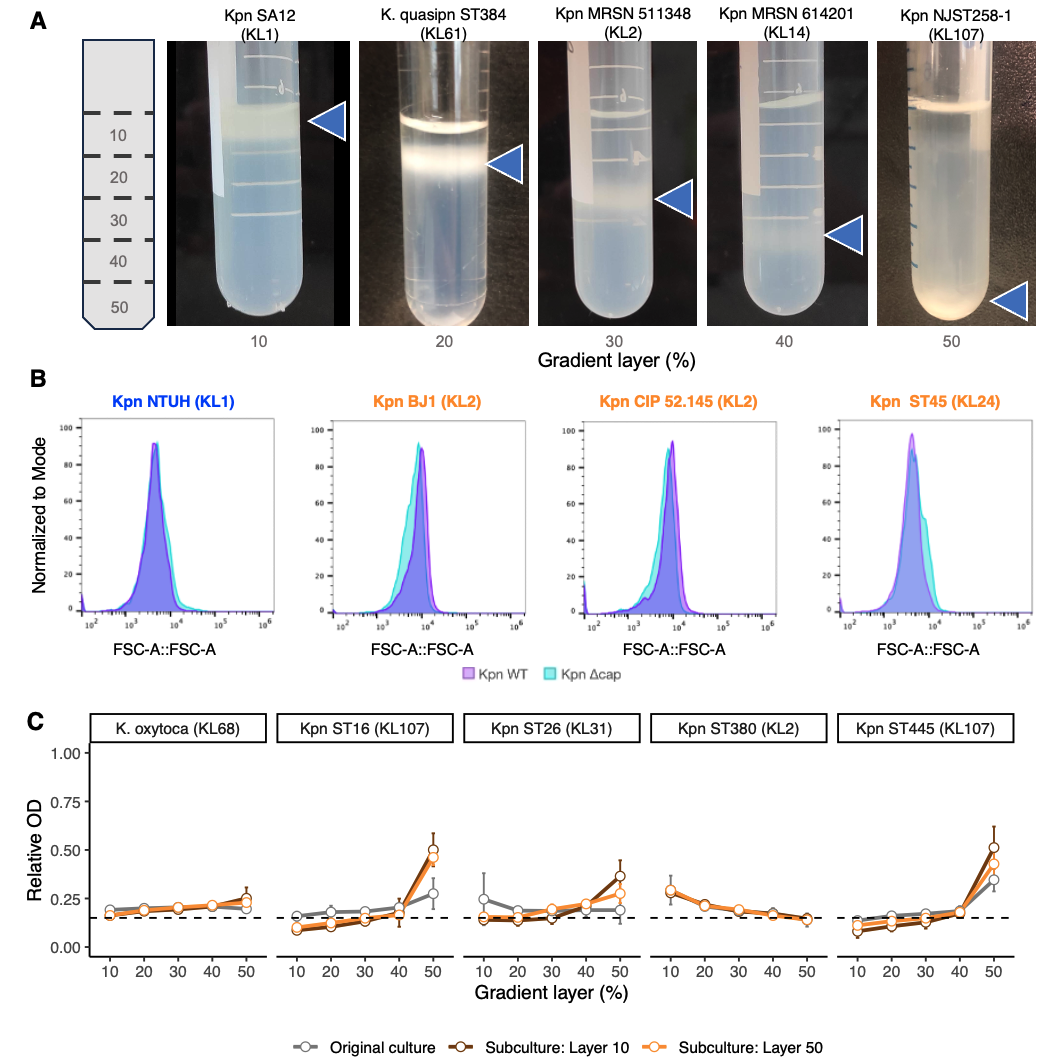


**Figure S1. Validation of phenotypic heterogeneity strategy in capsule production in *Klebsiella pneumoniae*. A.** Homogeneous strains with cells retained at the five different gradient layers. **B.** Flow cytometry histogram plots overlay showing the size of cells (FCS-A: Forward Scatter Area signal) for one non-heterogeneous *Kpn* strain (in blue, Kpn NTUH) and three heterogeneous strains (highlighted in orange) and their respective non-capsulated mutant strains as controls. The y-axis corresponds to the normalized percentage of cells. WT strains are indicated in purple and non-capsulated (∆cap) strains in light blue. A representative plot from one experiment is shown. **C.** Relative OD of several cultures initiated from different subpopulations. Each culture was separated by capsule production using a Percoll gradient (grey lines). The top layer (10%) containing hypercapsulated cells and the bottom layer (50%) harboring hypocapsulated cells were used to reinitiate two independent cultures. After growth, the new cultures were tested for heterogeneity. Error bars represent standard deviation.

**Figure S2.** **Visualization of capsule heterogeneity**. Overnight cultures of three different *Klebsiella* strains (Population), which exhibit phenotypic heterogeneity in capsule production, were visualized using a **light microscope** with oil immersion. The capsule appears as a white halo around the cell due to India ink staining. Cells were then separated by centrifugation across a Percoll gradient, and different layers were imaged again. Representative images are shown. Scale bar: 5 μm.
