# Supplementary material for: Phenotypic heterogeneity of capsule production across opportunistic pathogens
Source: mBio. 2025 Sep 4;16(10):e01807-25. doi: 10.1128/mbio.01807-25 (PMC12505892; doi:10.1128/mbio.01807-25)
Supplement: Supplemental Figures, Part 2 — Figures S3 to S6. [file mbio.01807-25-s0002.docx]

# SUPPLEMENTARY MATERIAL for

## Bet hedging of capsule production across opportunistic pathogens

Amandine Nucci^1#^, Julie Le Bris^1,2#^, Sara Diaz Diaz^3#^, Lilibeth Torres-Elizalde^3^, Eduardo P.C. Rocha^1^ and Olaya Rendueles*^1,3^

^1^Institut Pasteur, Université Paris Cité, CNRS UMR3525, Microbial Evolutionary Genomics, Paris 75015, France.

^2^Sorbonne Université, Collège Doctoral, École Doctorale Complexité du Vivant, 75005 Paris, France

^3^Laboratoire de Microbiologie et Génétique Moléculaires (LMGM), CNRS UMR5100, Centre de Biologie Intégrative (CBI), Université de Toulouse, CNRS, Université de Toulouse, Toulouse, France

# equal contribution

*Corresponding author, olaya.rendueles-garcia@utoulouse.fr


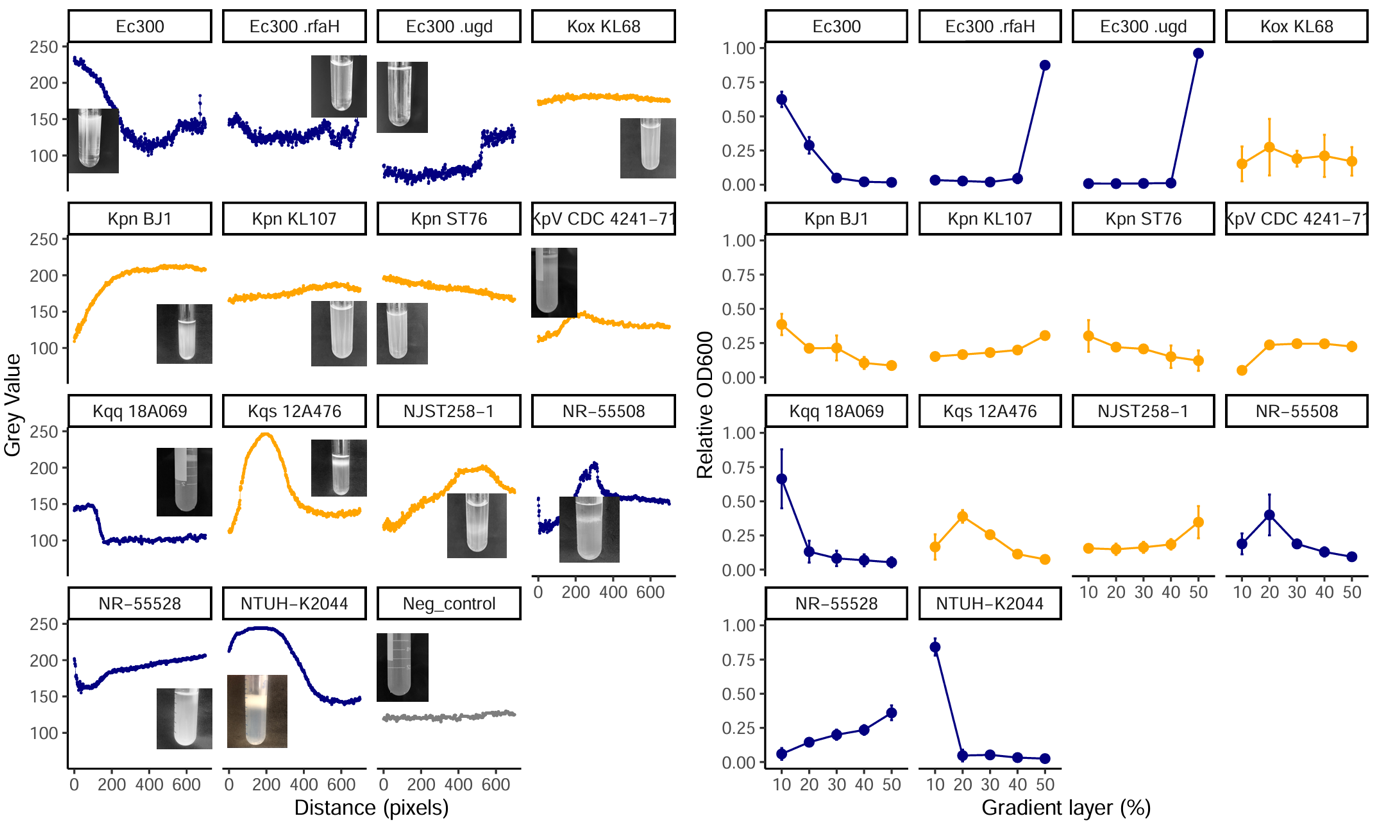


**Figure S3. Image analyses of several strains upon centrifugation in a Percoll gradient.** Pictures of each strain were taken and analyzed by ImageJ (see Methods). Grey values (y-axis) reflect the cell density along the tube, as measured by the distance from the top of the culture in pixels (x-axis). Insets show representative pictures for each culture. We also show the plots corresponding to the absorbance method (average across at least three biological triplicates).

**Figure S4. Method validation. A.** Distribution of the number of strains and the number of fractions with cells above the different relative OD cut-offs (0.05,0.1,0.15 and 0.2). Dashed line indicates the minimum of cells recovered in at least three different layers. Numbers on the top reflect the number of heterogeneous strains identified by each method. **B.** Heterogeneous strains and non-heterogeneous strains depending on the cut-off applied. Dotted line indicates cut-off value. The green lines indicate strains that were considered heterogeneous using a 0.1 cut-off but non-heterogeneous using a 0.15 value. **C.** Distribution of the number of strains and their inferred slopes, as obtained from linear regressions (GLM: Relative OD_600_ ~ gradient layer). **D.** Strains ordered by the slope and identified as heterogeneous using a 0.15 cut-off. Error bars indicate standard deviation of the mean slope from a minimum of three independent biological replicates. Dashed lines indicate slope values used to determine heterogeneity. **E.** Distribution of the Shannon entropy measure of heterogeneity and identified as heterogeneous using a 0.15 cut-off. **F.** Venn Diagram of all strain-environment combinations that were considered to be heterogeneous by at least one of the three different methods (N= 361). Heterogeneity of each strain-environment combination was calculated using the average of at least three independent replicates for each strain in each environment. Performing the same analyses with each individual biological replicate does not qualitatively alter the results.

**Figure S5. Phenotypic heterogeneity across isolates.** Examples of the diversity of phenotypes observed upon slow centrifugation in Percoll gradients. Examples portray different strains from different isolations sources (indicated in parenthesis). The shape of the data points indicates the growth conditions (Environment), as detailed in the legend.


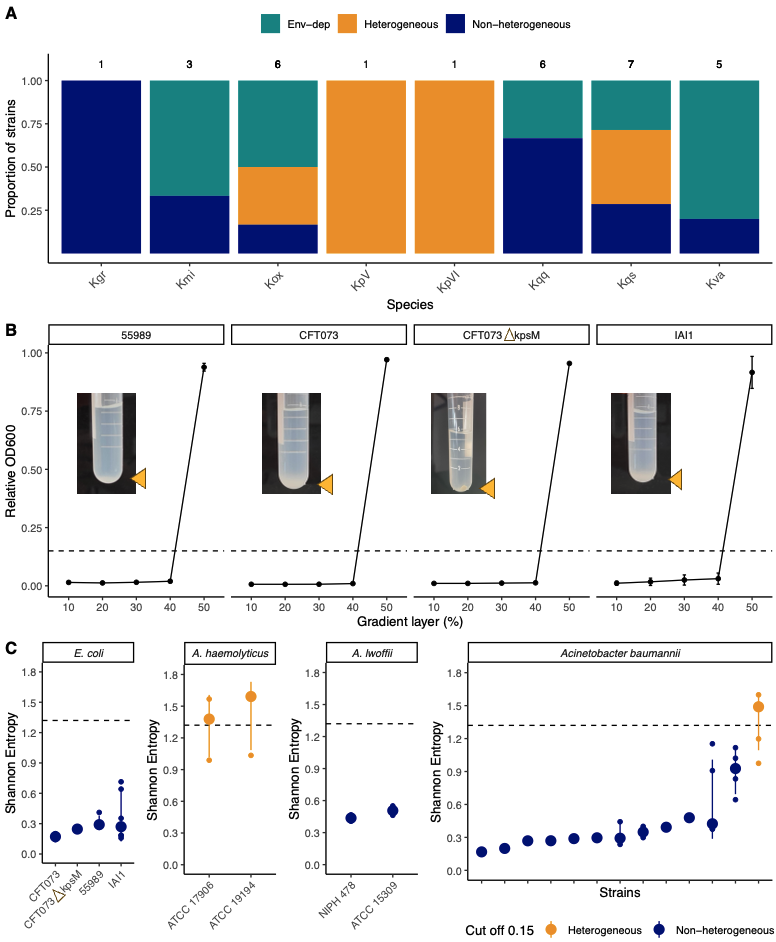


**Figure S6. Heterogeneity in other *Klebsiella* spp. strains. A.** Proportion of strains, other than *K. pneumoniae*, that are either heterogeneous, non-heterogenous, or dependent on the environment in which they were grown. (Kgr: *K.* *grimontii*; Kmi: *K. michiganenesis*; Kox; *K. oxytoca*; KpV; *K. variicola subs tropica*; KpVI: *K. varicola subs quasivariicola* Kp6; Kqq: *K. quasipneumoniae subsp quasipneumoniae;* Kqs: *K. quasipneumoniae subsp similpneumoniae; Kva: K. variicola).*
