# Supplementary material for: Phenotypic heterogeneity of capsule production across opportunistic pathogens
Source: mBio. 2025 Sep 4;16(10):e01807-25. doi: 10.1128/mbio.01807-25 (PMC12505892; doi:10.1128/mbio.01807-25)
Supplement: Supplemental Figures, Part 3 — Figures S7 and S8. [file mbio.01807-25-s0003.docx]

# SUPPLEMENTARY MATERIAL for

## Bet hedging of capsule production across opportunistic pathogens

Amandine Nucci^1#^, Julie Le Bris^1,2#^, Sara Diaz Diaz^3#^, Lilibeth Torres-Elizalde^3^, Eduardo P.C. Rocha^1^ and Olaya Rendueles*^1,3^

^1^Institut Pasteur, Université Paris Cité, CNRS UMR3525, Microbial Evolutionary Genomics, Paris 75015, France.

^2^Sorbonne Université, Collège Doctoral, École Doctorale Complexité du Vivant, 75005 Paris, France

^3^Laboratoire de Microbiologie et Génétique Moléculaires (LMGM), CNRS UMR5100, Centre de Biologie Intégrative (CBI), Université de Toulouse, CNRS, Université de Toulouse, Toulouse, France

# equal contribution

*Corresponding author, olaya.rendueles-garcia@utoulouse.fr

.

**Figure S7. Phenotypic heterogeneity is dependent on the environment.** Heterogeneous (**A**) and non-heterogeneous (**B**) strains across all environments. **C.** Proportion of heterogeneous strains in function of the environment and their capsule locus types. The numbers on top of bars indicate the amount of strains analyzed. We only considered capsule locus types for which we possessed at least three different strains.

**Figure S8. Different patterns of heterogeneity profile across environments.** Strain NTUH-K2044 is non-heterogeneous in all environments, with most cells accumulating in the upper gradient layer, indicative of hypercapsulation. NR-55600 shows no heterogeneity and is hypocapsulated. Strain CIP 52.208 is also non-heterogenous, but there is a shift in the amount of capsule production in nutrient-poor environment (M02). Strain NR-55597 is heterogenous in all environments. Kpn ST76 is heterogeneous in all environments except in nutrient-poor media, the opposite is true for NR-55516. Error bars indicate standard deviation (at least 3 independent biological replicates).
