# Supplementary material for: Phenotypic heterogeneity of capsule production across opportunistic pathogens
Source: mBio. 2025 Sep 4;16(10):e01807-25. doi: 10.1128/mbio.01807-25 (PMC12505892; doi:10.1128/mbio.01807-25)
Supplement: Supplemental Figures, Part 4 — Figures S9 to S13. [file mbio.01807-25-s0004.docx]

# SUPPLEMENTARY MATERIAL for

## Bet hedging of capsule production across opportunistic pathogens

Amandine Nucci^1#^, Julie Le Bris^1,2#^, Sara Diaz Diaz^3#^, Lilibeth Torres-Elizalde^3^, Eduardo P.C. Rocha^1^ and Olaya Rendueles*^1,3^

^1^Institut Pasteur, Université Paris Cité, CNRS UMR3525, Microbial Evolutionary Genomics, Paris 75015, France.

^2^Sorbonne Université, Collège Doctoral, École Doctorale Complexité du Vivant, 75005 Paris, France

^3^Laboratoire de Microbiologie et Génétique Moléculaires (LMGM), CNRS UMR5100, Centre de Biologie Intégrative (CBI), Université de Toulouse, CNRS, Université de Toulouse, Toulouse, France

# equal contribution

*Corresponding author, olaya.rendueles-garcia@utoulouse.fr


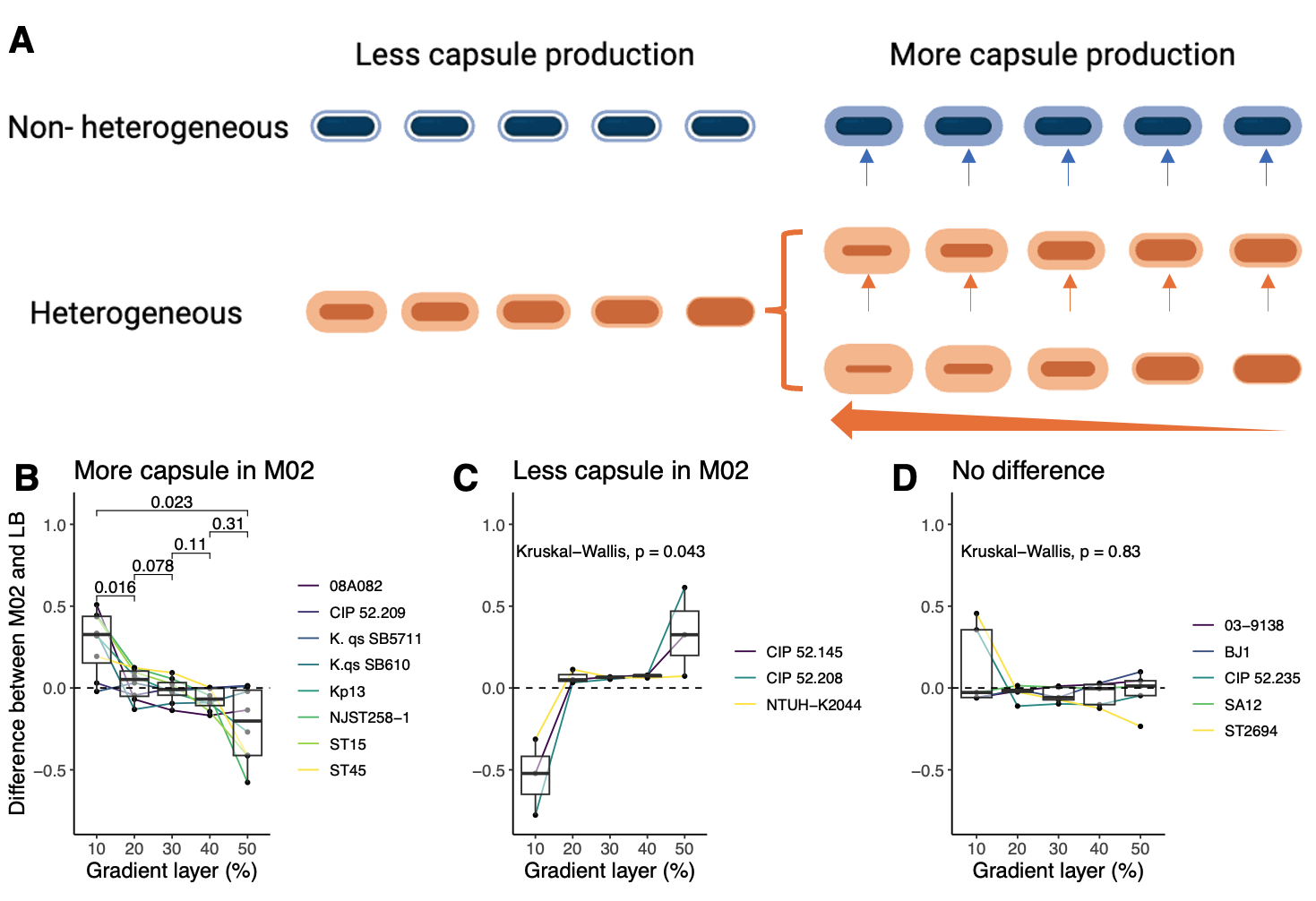


**Figure S9. Changes in population composition in nutrient-rich and nutrient-poor growth media.** **A.** Diagram of how changes in capsule production at the population level can be achieved. Non-heterogeneous populations can only increase capsule production by generic transcriptional upregulation, which results in a homogeneous increase across all cells. Heterogeneous populations can increase capsule production as non-heterogeneous populations. However, these populations can also shift the proportion of hypercapsulated and hypocapsulated cells. Thus, when more capsule is produced in nutrient poor media (M02) (**B**), the proportion of cells in the upper gradient layer (10%) is expected to be greater than in nutrient rich media (LB). Consequently, we observe more hypercapsulated cells and less hypocapsulated cells. Lines link values for each independent strain. Values above zero correspond to cases in which more cells are found in nutrient poor than in nutrient rich media. Panels are divided by instances in which strains produce more (**B**), less (**C**) or equivalent (**D**) capsule in nutrient-poor (M02**)** and nutrient-rich (LB) media at the population level. Raw data from Buffet et al. (33) were used to identify strains which produced more, less or similar capsule amount at the population level across nutrient-rich and nutrient-poor environments.

**Figure S10. Controls for capsule exchanges**. We observe that all non-capsulated strains pellet and are found in the last gradient layer (50%). All wildtype strains and their respective control swaps (complemented strains) have the same heterogeneity phenotype.

**Figure S11. India ink staining images of *Kpn* NTUH K2044 (A), *Kpn* BJ1 (B), and their respective capsule exchanges to *Kpn* NTUH K2044 K24 (C) and *Kpn* BJ1 K1 (D).** Images were taken at a magnification of 1000× after an overnight culture in LB. Scale bar = 10 μm. Capsule thickness is determined by the exclusion area around cells (negative staining) due to the capsule.

**Figure S12. Fitness of heterogeneous and non-heterogeneous strains in LB.** Capsulated strains were competed against their respective-non capsulated strains in an initial 1:1 ratio. The fitness of the capsulated strain is displayed. Each dot represents the mean of at least three independent replicates, error bars indicate the standard deviation from the mean. Raw data from competitions from Buffet et al. (33).

**Figure S13. *E. coli* and *Acinetobacter* spp*.* phenotypic heterogeneity in capsule production***.* **A.** *E. coli* strain expressing a group 2 capsule (*E. coli* CFT073), and its non-capsulated mutant due to a deletion of *kpsM,* essential for capsule production, as well as two strains expressing group 1 capsule (*E. coli* IAI1 and *E. coli* 55989) were tested for capsule heterogeneity. Strain Ec300 encoding a *K. variicola* group 1 capsule and two different non-capsulated mutants (∆*rfaH* – positive capsule regulator, and ∆*ugd* (also noted as ∆1040 in the manuscript of origin(42))– an essential capsule gene) were also tested for phenotypic heterogeneity. The dashed line corresponds to the 0.15 cut-off. If at least three gradient layers have a relative OD_600_ above this threshold, the strains are considered heterogeneous. **B**. Microscopic images of *E. coli* Ec300 expressing a horizontally transferred capsule from a *K. variicola* strain, a non-capsulated mutant, as well as *E. coli* CFT073 and its non-capsulated mutant (∆*kpsM*). The thin capsule of CFT073 cannot be observed by India ink, and photographic images do not allow to distinguish the wild type from its non-capsulated mutant. Scale bar of 10 μm is represented. **C.** Pictures of strains showing visible differences between capsulated and non-capsulated strains, but also between lightly capsulated CFT073 and heavily capsulated Ec300. **D.** Shannon entropy of *E. coli* and *Acinetobacter* spp. The dashed line corresponds to the established value of 1.3, above which the samples are considered heterogeneous according to the entropy measurement. Colors indicate heterogeneity according to the cut-off method, using a threshold of 0.15. Small dots represent individual replicates, the larger dot corresponds to the median, and error bars are standard deviations from the median.
