# Supplementary material for: Phenotypic heterogeneity of capsule production across opportunistic pathogens
Source: mBio. 2025 Sep 4;16(10):e01807-25. doi: 10.1128/mbio.01807-25 (PMC12505892; doi:10.1128/mbio.01807-25)
Supplement: Supplemental Material — Supplemental text, Table S3, and supplemental table captions. [file mbio.01807-25-s0006.docx]

# SUPPLEMENTARY MATERIAL for

## Bet hedging of capsule production across opportunistic pathogens

Amandine Nucci^1#^, Julie Le Bris^1,2#^, Sara Diaz Diaz^3#^, Lilibeth Torres-Elizalde^3^, Eduardo P.C. Rocha^1^ and Olaya Rendueles*^1,3^

^1^Institut Pasteur, Université Paris Cité, CNRS UMR3525, Microbial Evolutionary Genomics, Paris 75015, France.

^2^Sorbonne Université, Collège Doctoral, École Doctorale Complexité du Vivant, 75005 Paris, France

^3^Laboratoire de Microbiologie et Génétique Moléculaires (LMGM), CNRS UMR5100, Centre de Biologie Intégrative (CBI), Université de Toulouse, CNRS, Université de Toulouse, Toulouse, France

# equal contribution

*Corresponding author, olaya.rendueles-garcia@utoulouse.fr

Table of contents

[SUPPLEMENTARY TEXT 2](#_Toc200529978)

[SUPPLEMENTARY TABLES 3](#_Toc200529979)

## SUPPLEMENTARY TEXT 1

**Image-Based Analyses of Percoll Gradients**

To complement optical density measurements of the Percoll gradients, we developed an image-based method to assess capsule heterogeneity in *Klebsiella* strains. We took standardized photographs of the culture tubes immediately after slow centrifugation. These photographs were converted to a gray scale using ImageJ, and the pixel intensity along the vertical axis of the tube was measured. The resulting intensity profiles reflect the distribution of cells across the gradient with darker regions corresponding to increased cell density (higher absorbance). In non-heterogeneous samples, a clear unimodal peak could be observed, whereas in heterogeneous strains the distribution was much broader (Figure S3). The image-derived intensity curves correlate with absorbance readings of the independent layers of the gradient. This constitutes a proof-of-concept that image analyses can be used as a simple, fast alternative for evaluating heterogeneity. Further, this could be amenable for higher throughput screens without additional pipetting and absorbance measurements.

## SUPPLEMENTARY TABLES

**Table S1. *Klebsiella pneumoniae* strains used in this study (Supplemental Dataset 1).**

**Table S2. Strains belonging to other species used in this study (Supplemental Dataset 2).**

**Table S3. General linear model was used to test an association between heterogeneity and virulence score.** The general linear models were calculated with the function glm() from the base package of R, and the link function used was binomial. The virulence score was calculated by Kleborate V2.0.

Specifically, the formula used was:

Heterogeneity (0/1) ~ Virulence score (0-5), family = binomial(link = "logit")

The null deviance: 250.52 on 181 degrees of freedom, and the residual deviance: 246.24 on 180 degrees of freedom

AIC: 250.24

Number of Fisher Scoring iterations: 4

|  | Estimate | Standard Error | Z-value | P-value |
| --- | --- | --- | --- | --- |
| Virulence Score | -0.249 | 0.126 | -1.979 | 0.047 |
| Intercept | 0.008 | 0.175 | -0.048 | 0.962 |

**Table S4. Heterogeneity data.** All strains tested in the different environments. Heterogeneity was determined by three independent methods; using the slope of the general linear model (GLM), a cut-off (CutOff) of 0.15, by which at least three different gradient layers have 15% of the cells, and Shannon diversity index (Shannon).

**Table S5. Mutant strains used in this study.** List of mutants used in this study alongside key characteristics as well as the experiments and figures in which they were used.
